# Predictive ability of underlying factors of motorcycle rider behavior: an application of logistic quantile regression for bounded outcomes

**DOI:** 10.15171/hpp.2017.40

**Published:** 2017-09-26

**Authors:** Masoumeh Babajanpour, Mohammad Asghari Jafarabadi, Homayoun Sadeghi Bazargani

**Affiliations:** ^1^Department of Statistics and Epidemiology, Faculty of Health, Tabriz University of Medical Sciences, Tabriz, Iran; ^2^Road Traffic Injury Research Center, Tabriz University of Medical Sciences, Tabriz, Iran

**Keywords:** Logistic quantile regression, Bounded outcomes, Motorcycle traffic accidents, ADHD, MRBQ

## Abstract

**Background:** The human factors are of great importance, especially Motorcycle Rider Behavior Questionnaire (MRBQ) and attention deficit hyperactivity disorder (ADHD) in motorbike riders in road traffic injuries. This study aimed to predict MRBQ score by ADHD score and the underlying predictors by the logistic quantile regression (LQR), as a new strategy.

**Methods: **In this cross-sectional study, 311 motorbike riders were randomly sampled by a clustering method in Bukan, northwest of Iran. The data were collected by MRBQ and ADHD standard surveys. To assess the relationship at all levels of MRBQ distribution, LQR in 5th, 25th, 50th, 75th and 95th quantiles of MRBQ score was utilized to assess the predictability of ADHDscore and its subscales in addition to the underlying predictors of MRBQ score. To do this, an unadjusted and as well as adjusted 4-step hierarchical modeling was used.

**Results:** Almost in all quantiles of MRBQ scores, direct and significant relationships were observed between MRBQ score and ADHD score and its subscales (coefficients: 0.02 to 0.10, all P < 0.05). Besides, the driving period (coefficients: -0.58 to -0.95, P < 0.05) and hour driving (coefficients: 0.42 to 0.52, P < 0.05) also came to be the significant predictors of MRBQ score.

**Conclusion: **ADHD score and driving parameters can be taken into the consideration when planning actions on the motorcycle rider behaviors at all levels of the MRBQ.

## Introduction

Road traffic injury is a global problem. Based on a report by the World Health Organization (WHO), road traffic injuries kill 1.27 million individuals annually and 20-50 million people are injured subsequently.^1^ As well; the accidents are the ninth causes of mortalities in the world and the first cause of mortalities among youth aged 15 to 29.^2^ It is predicted that the accidents would be the seventh cause of mortality in 2030.^3^ According to the WHO reports, the mortality rate of traffic accidents is higher than Iran in only 4 countries. Iran has less than 1% of world’s population whilst it has more than 2.5% of world traffic accidents.^4^ Although, in recent years, the mortalities by road traffic injury have decreased in Iran, still it has been ranked as the third cause of mortality after cardiovascular disease and stroke.^5^ The reports showed that death rate from road accidents in Iran is 20 times higher than the global average.^6^

Motorcycle drivers are among vulnerable groups in road accidents,^7^ in a way that, compared with car drivers. They have 8, 4 and 2 times higher risk of death, the risk of injury and risk of having an accident by the pedestrians respectively.^8^ A motorcycle has 9.3 times more possibilities of an accident than the cars.^9^ Compared to high-income countries. In low- and middle-income countries, the great parts of the population are pedestrians, bicycle and motorcycle riders and 90% of happened mortalities from traffic accidents in these countries.^10^ In Iran, more than 51% of traffic accidents are involving motorcyclists.^11^

According to the WHO, road deaths comprise 25% of all deaths caused by injuries. Human agent is the main cause in 60% of vehicle accidents.^12,13^ Among the human agents, cognitive attention, as one of the most important aspects, is the main causes of traffic accidents in a way that it comprises 20%-50% of accidents.^14^ In this regard, one factor that contributes to road traffic injuries and accidents are attention deficit hyperactivity disorder (ADHD) which is a developmental chronic nervous disorder. Those individuals with this disorder experience significant difficulties in various aspects to their lives. As a factor, motorcyclist driving behavior is the last link to the chain of human causal and psychological factors to the accident.^15,16^ When examining the undeniable role of humans in the chain of events leading to the accident, the identification of this factor can be one valuable action in traffic safety.^17^ The first study about hyperactive drivers and road safety was carried out by Weiss et al.^18^ The landmark of the studies about ADHD and road safety, was taken place by Barkley et al.^19^ They showed that drivers with ADHD had three to 4 times higher odds of an accident than drivers without ADHD.^19^ Sadeghi-Bazargani et al studied the relationship between motorcyclists’ behavior and ADHD with motorcycle traffic injuries by common binary logistic model. They showed that riding behavioral scale and ADHD subscale B scored by age, educational degree, and the reason for motorcycle riding could be considered as potential determinants of motorcycle injuries.^20^

Considering the importance of human health in medical and epidemiologic studies, the accuracy of the results is more important, therefore; those statistical models that have the minimum bias and error should be utilized. Applying statistical models without this criterion may not be tailored to such data and may lead to bias in results and decision making.^21^ All previously analyzed data done on MRBQ were based on generalized linear models (GLMs).^20,22,23^ which did not take into the account the limitation of the bounded nature of variable, and may lead to bias in findings.^24^ Specific statistical methods are required to comprehensively address the causes and risk factors of major road traffic accidents and their consequences. Therefore, the present investigation aimed to utilize logistic quintile regression (LQR) to investigate the predictors of motorcycle behaviors (assessed by MRBQ). Unlike partial/average partial/average partial/average view of the relationship that classical statistical models present and as an advantage, this model provides a description of the relationship at different points of the outcome. Using various quantiles in response instead of just mean response and implementing the bounded nature of the outcome, the LQR could provide more comprehensive projections of risk factors of MRBQ.

## Materials and Methods

### Participants and procedures

A total of 311 Iranian motorcycle ridermen recruited in this cross-sectional study based upon a cluster sampling scheme in Bukan, northwest Iran in 2016. Bukan is located in west Azerbaijan province. The population of this city consists of 224 628 persons according to the general census of Iranian statistical center in 2011. Bukan city was divided into 14 homogeneous clusters, and then 7 clusters were randomly chosen. Afterward, enough samples were collected in each cluster to achieve the determined sample size. Data were collected through referring homes and motorcycles shops. Some adaptations were done with sampling design and sample selection for feasibility to perform sampling. The inclusion criteria were used motorcycle (at least 3 times per month), age +15 years, residing in Bukan and being conscious and alert when filling out the questionnaire. The exclusion criteria were a lack of motivation to participate and to complete the questionnaires in a self-descriptive manner.

The study size was determined using primary information obtained from the study by Sadeghi-Bazargani et al^20^ on the main outcome of this study, the relation between MRBQ and ADHD. Considering 95% confidence level and 80% power, the sample was estimated to be 227 subjects according to odds ratio (OR) about 1.4 as the effect size. Taking into account the cluster design, the sample size was increased to 296 cases by a design effect of 1.3 and then increased to 311 for more precision.^20^

### Study variables and measurements

The study main variables included Motorcycle Rider Behavior Questionnaire (MRBQ) as the outcome and ADHD as the predictor of MRBQ. Data were collected in a self-descriptive manner using MRBQ (with 48-items) and Conner’s short-form ADHD questionnaires to assess the motorcycle riders’ behaviors and ADHDs respectively.

In the present investigation, the MRBQ was utilized to assess motorcycle riders’ behavior as the outcome variable. MRBQ was first built in 2007 by Elliott et al^25^ and developed by Ozkan et al.^26^ In this study the internal consistency reliability, as assessed by Cronbach’s alpha, was supported for MRBQ (α = 0.896). The respondents were asked to report the frequency of their behaviors during last year by selecting one of the 5 points scales (0 = never, 1 = hardly ever, 2 = occasionally, 3 = quite often, and 4 = nearly all the time). The MRBQ score was computed by summing over the items. The score ranged over 0-192 where in the higher scores indicate the less attention to the traffic rules.

The ADHD questionnaire was also translated, and the validity and reliability were assessed and confirmed in a study by Sadeghi-Bazargani et al.^27^ In this research, the internal consistency reliability was supported for ADHD scale (α = 0.891) and for all ADHD subscales (0.643-0.899). ADHD has 4 subscales; subscale A measuring inattention (I1 +I9 +I13 +I14 +I19 +I21 +I26 +I29 +I30), subscale B measuring hyperactivity, impulsivity (I2 + I4 + I6 + I8 + I16 + I18 + I22 +I25 +I27), subscale C (A +B), and subscale D measuring ADHD index (I3 +I5 +I7 +I10 +I11 +I12 +I15 +I17 +I20 +I23 +I24 +I28). The symptomatology of the scale is based on the DSMIV (Diagnostic and Statistical Manual of Mental Disorders, Fourth Edition), which the diagnostic criteria for ADHD are similar to those in the DSMV.^28^ Rating scales will ask the respondents to score behaviors on a 4-point frequency scale ranging from 0 = never/rarely to 3 = very often. ADHD scale and all subscales scores were computed by summing over the items (ranges over 0-90 for total score 0-27 for A and B subscales, 0-54 for C subscale and 0-36 for D subscale). The higher the score, the more severe the symptom is.

The predictors of MRBQ in this study were considered to be: age, marital status, educational level, job status, income level, house price level, car price level, hygiene cost level, motorcycling aim, using helmet, having driving license, driving license period, driving period, hour driving, number of days used, sub-accident, vehicle type, cell phone answering.

To completely take into the account the above-mentioned predictors, both trend effect, and effect compared to a reference category were considered as the LQR models. When a complete set of quintiles showed a significant relationship with outcome variable MRBQ, then the effect considered to be significant. The missing values were deleted listwise and since they were less than 5%, the effect of missing values was ignorable.

### Statistical Analyses

Data were summarized and presented using the mean (SD) or the median (percentile 25 - percentile 75) along with (Minimum-Maximum) for numeric and the frequency (percent) for categorical variables, respectively.

The data on MRBQ outcome (ranged over 0-192) was projected by LQR via the following equation:

Logit(MRBQ) = log[(MRBQ + Ɛ)/(192 – MRBQ + Ɛ)]

We considered Ɛ = 0.001. And finally our model was:

*Q_logit(MRBQ)_(P) = β_P,0_ + β_P,1_ADHD*

ADHD is a predictor of MRBQ in this model. The bounds were set as y_min_ = -0.001 and y_max_= 192.001 and the 5 quantiles of *P* = 0.05, 0.25, 0.5, 0.75 and 0.95 were considered. Furthermore, the standard error and *P* values were estimated using 1000 bootstrap samples. The model parameters were estimated using lqreg package in Stata, utilizing the codes lqreg, lqregpred and lqregplot and then the bootstrap standard errors are estimated.^24,29^ Based on LQR model and considering *P *< 0.1 in the univariate analyses for all quantiles, the variables were chosen to enter into the multivariate model which includes age, income, marital status, car price, using a helmet, years of driving records, days driving, driving hours, answering a cell phone, number of cars, ADHD and ADHD subscales. In the multivariate modeling strategy, 4 models were fitted taking considering *P *< 0.05. In model 1, the MRBQ was modeled with significant background variables in the univariate analyses. In the model 2, the ADHD score was added in the model 1. In model 3, the subscales of BSS and ASS of ADHD were added in the model1. Finally, in the model 4, the subscale DSS was added in the model 1. In each model confidence interval and *P* values were computed for *P *= 0.05, 0.25, 0.50, 0.75, and 0.95. In addition to the analyses done by indicator categorical variables, trend analyses were done by directly entering the ordinal categorical variables to the models. All analyses were performed using STATA 14 software (Stata Corp., College Station, Texas 77845 USA).

## Results

A total of 311 subjects participated in this study. A number of 104 persons had age between 25 to 28 years. 21.22%, 45.34% and 33.34% of samples had primary, diploma and university degree, respectively. Most of the subjects (81.03%) had not a driving license. 71. 38% had more than 2 years of driving record, and 77.49% had not an accident in their driving lifetime. Other information on background variables are presented in Table 1.

The summarized measure of MRBQ outcome and the main predictor, i.e. ADHD and its subscales are presented in Table 2. The results show that in MRBQ and ADHD scores and their subscales were less than the possible average score could be obtained as showed in Table 2.

Based on LQR model, considering *P *< 0.1 in the univariate analyses for all quantiles of MRBQ score, the significant variables went back to ADHD score, and all ADHD subscales score beside the marital status, using a helmet, years of driving records, driving hours, days driving, cell phone answering. Furthermore, ADHD all its subscales scores besides the age, income, car price, years of driving records, driving hours, and the number of cars, were significant in the trend analyses (Table 3).

The modeling results in the multivariate analyses are presented in Table 4. In model1, considering the background variables in the predicting MRBQ, the marital status, driving period and driving an hour were significant. In model 2, considering the relationship of ADHD with MRBQ, controlling for the variables in the model 1, the ADHD score was significantly related with MRBQ in the adjusted model for almost all levels (quantiles) of MRBQ score. In model 3, controlling for the variables in model 1, the relationship of BSS and ASS subscales were significant for some levels of MRBQ score. In model 4 the relationship of the DSS subscales was significant in the adjusted model for almost all levels of MRBQ score, controlling for variables in model 1.

## Discussion

The present research demonstrated the predictive ability of underlying factors of motorcycle rider behavior utilizing LQR. Regarding the MRBQ prediction by ADHD and the other underlying factors, the findings showed that in univariate modeling decrease in age, income and driving days were related to increasing in MRBQ while increasing in ADHD and its subscales and driving hours were related to increasing in MRBQ. Besides that, marital status, income, driving, cell phone answering were significant and entered into the multivariate model.

In the multivariate modeling for previously mentioned variables in the univariate modeling, it can be said that increase in ADHD and all its subscales went back to the increase in MRBQ. The results showed a stronger relationship between DSS subscale compared with BSS and ASS subscales, in which DSS was significantly related in more quantiles compared to BSS and ASS. Our results were in the line with Sadeghi-Bazargani and colleagues’ study that by multivariate analysis; they found the relationships between ADHD and MRBQ and then with motorcycle injuries were significant with a different pattern for ASS and BSS subscales Sadeghi-Bazargani et al, in their study, found a relationship between ADHD and MRBQ. They showed that BSS and ASS subscales were significantly related to MRBQ in various modeling.^20^ The similarity between two studies may be due to similar study population in which the Iranian drivers show the same behaviors. The Canadian driving center obliged controlled ADHD as an item to pass the driving test.^30^ Another study showed that training safe driving behavior, training driving techniques and skills and how to drive vehicle in different situations and utilization of planned behavior approach theory can change the people’s attitude and can be regarded as a very influential variable on safety driving. This issue eventually provides a setting to decrease traffic risks and physical injury by using safety equipment. Behavioral training is necessary to control dangers caused by the beginners and those drivers with ADHD.^31^ As researches say, those with ADHD are usually more willing to take risks while most of these risks are conscious. This subject should be considered as a risky behavior because it is responsible for about 25% to 30% of road accidents in Iran.^20^

Furthermore, we found that MRBQ distribution in single and married individuals was identical except for 95th quantile of MRBQ, which was significantly greater in married than single participants in the adjusted models. Also, nearly in all models, the driving period showed a significant and direct relationship with almost all quantiles of MRBQ score. Besides that, by considering the relationship between MRBQ and using a cell phone and some behavioral violations, the results of our study can be in the line with other studies.^20,27^ Moreover, significant relationships between injury outcome and age, education level, marital status and the type of intention to drive motorcycles.^20^

Our rationale in the application of LQR in describing the relationship between underlying predictors of a bounded outcome MRBQ were:

1. The LQR represents a useful methodology to extrapolate the conditional distribution of bounded outcomes giving a set of risk factors. The results are valid in terms of any basic distribution, and the predictions for the outcome are limited within the bounded range.^24^
2. By assessing the sets of quantiles, LQR provides a thorough comparison of population distributions with respect to location, spread, and any other features. Generally, LQR allows more sound understanding than any other technique that considers the only single summary measures, such as median or the mean.^24^ LQR was utilized by other studies to model the bounded outcome.^24,32,33^

### Study limitations

We limited the modeling by using the logit link in this study as used by other studies utilized the LQR in the modeling of bounded outcomes.^24,33^ The logit link function is a proper and simple transformation of the prediction curve. It also provides odds ratios. These 2 features have made it popular among researchers. In the future studies, instead, it is suggested to take into account the modeling of such outcomes using probit link function, predicting the underlying latent variable and log-log complementary link function for extreme asymmetric distributions.^34^ Additionally, beta regression and boosted beta regression models can be suggested in this setting; they interpret the parameters in terms of the mean of bounded outcome and are unsurprisingly heteroskedastic and easily accommodate asymmetries.^35,36^ MRBQ has a wide range and despite the bounded nature which encounters the linear regression model with the structural problem of non-equity of the 2 sides of the equations. However, the underlying assumptions of the linear regression were mated in our data, and we shift to LQR because of the structural problem.

Other limitations were the self-descriptive nature of the questionnaires which are common in such studies. The data were limited to a sample of motorcycle riders in a small city in the northwest of Iran, which may not be generalizable to other parts of Iran due to different patterns of behavior.

Additionally, the model may perform more optimally in the more limited bound of the outcome variable. And this issue is recommended to be studied in the future.

## Conclusion

The present investigation demonstrated the application of LQR in describing ADHD, its subscales and underlying predictors of the MRBQ as a bounded outcome. Considering the predictive ability of ADHD and its subscales as well as age, income, driving, days of driving, hours of driving, marital status, income, driving and answering the cell phone for MRBQ, that potentially caused road traffic injury among motorcyclists, all these factors can be useful and could be recommended for better planning and also designing educational programs by relevant organizations and policy makers.

## Ethical approval

The study protocol was approved by the ethical committee of Tabriz University of Medical Sciences (ethic code: TBAMED.REC.194.783). The participants were free to participate in the study, and the obtained information was just used for scientific purposes, and privacy was preserved meanwhile. All participants filled and signed the informed consent and assent. For the illiterate people, the informed consent form was read by the researcher or someone to whom he/she trusts. Then instead of signing, fingerprints were taken from participants.

## Competing interests

The authors declared no potential conflicts of interest with respect to the research, authorship, and/or publication of this article.

## Authors’ contributions

All authors read and approved the final manuscript. MAJ and HSB conceived of the study and participated in the design and data collection. MAJ and MB participated in the data analyses and MS preparation.

## Acknowledgments

We would like to appreciate Research Deputy of Tabriz University of Medical Sciences who supported our study.

**Table 1:** Descriptive characteristics of study’s participants and frequency and percent of MRBQ in each level of predictive variables

**Variables**	**No. (%)**
Job status	
Worker	90 (28.94)
Market	29 (9.32)
Service	45 (14.47)
Free	132 (42.44)
Government	15 (4.42)
Income level	
<600	40 (12.86)
600-1000	72 (23.15)
1000-1500	81 (26.05)
1500-2000	91 (29.26)
2000-5000	27 (8.68)
House Price level	
Not have	146 (46.95)
<100	63 (20.26)
100-200	72 (23.15)
>200	30 (9.65)
Car price level	
Not have	235 (75.56)
<10	48 (15.43)
>10	28 (9.00)
Hygiene cost level	
<10	94 (30.23)
10-20	135 (43.41)
20-40	64 (20.58)
>40	18 (5.79)
Motorcycling aim	
Recreation, journey, recreation & journey	219 (70.42)
Work & profession, work journey recreation	92 (29.58)
Using helmet	
Always	47 (15.11)
Often	59 (18.97)
Sometimes	59 (18.97)
Seldom	91 (29.26)
Never	55 (17.685)
Having driving license	
Have	89 (28.62)
Not have	222 (71.38)
Driving license period	
<1 year	10 (11.36)
1-3 years	24 (27.27)
3-5 years	21 (23.86)
>5 years	33 (37.50)
Driving period	
<1 year	59 (18.97)
< 2 days	252 (81.03)
Hours driving	
< 2 hours	29 (9.32)
2-5 hours	247 (79.42)
> 5 hours	35 (11.25)
Sub accident	
Have	70 (22.51)
Not have	241 (77.49)
Vehicle type	
Pedestrian	5 (7.25)
Motorcycle	9 (13.04)
Car	34 (49.28)
Lorry	21 (30.43)
Cell phone answering	
Always	49 (15.76)
Often	77 (24.76)
Sometimes	68 (21.86)
Seldom	63 (20.26)
Never	54 (17.36)
Number of days used	
<4 days	100 (32.16)
4-6 days	130 (41.80)
≥7 days	81(26.05)

**Table 2:** Summary statistics of main study variables (n = 311)

	**Mean**	**SD**	**Minimum**	**Maximum**
MRBQ score	63.3	22.8	0	126
ADHD score	27.8	12.6	0	66
ASS score	7.6	4.2	0	21
BSS score	9.1	4.1	0	19
CSS score	16.7	7.7	0	36
DSS score	11.1	5.4	0	30

Abbreviations: MRBQ, Motorcycle Rider Behavior Questionnaire; ADHD, Attention Deficit Hyperactivity Disorder; ASS score, subscale A measuring inattention; BSS score, subscale B measuring hyperactivity, impulsivity; CSS score, subscale C the sum of A and B subscales; DSS score, subscale D measuring ADHD index. MRBQ ranges over (0, 192) and ADHD ranges over (0, 90); ASS and BSS ranges over (0, 27); CSS ranges over (0, 54); DSS ranges over (0, 36).

**Table 3:** Relationship between underlying predictors of MRBQ and ADHD with MRBQ Outcome variable by LQR

Variables	**Percentiles**				
	**5**	**25**	**50**	**75**	**95**
	**B (90 CI),** ***P***	**B (90 CI),** ***P***	**B (90 CI), P**	**B(90 CI),** ***P***	**B(90 CI),** ***P***
Age	-0.09, (-0.31 to 0.14), 0.524	**-0.07, (-0.13 to -0.01), 0.083**	**-0.11, (-0.17 to -0.05), 0.003**	**-0.17, (-0.29 to -0.05), 0.019**	**-0.36, (-0.52 to -0.21), <0.001**
Marital status (married)	-0.17, (-0.78 to 0.43), 0.636	**-0.23, (-0.41 to -0.06), 0.030**	**-0.29, (-0.44 to -0.14), 0.002**	**-0.27, (-0.55 to -0.01), 0.100**	-0.32, (-0.87 to 0.23), 0.338
Education level					
Illiterate	Referent	Referent	Referent	Referent	Referent
Primary	**-0.91, (-1.70 to -0.10), 0.058**	-0.20, (-0.57 to 0.16), 0.361	0.25, (-0.19 to 0.69), 0.333	**0.69, (0.24 to 1.15), 0.012**	1.09, (-7.72 to 9.90), 0.838
Diploma	**-0.49,(-0.78 to -0.20), 0.006**	-0.13, (-0.37 to 0.10), 0.341	**0.22, (0.01 to 0.44), 0.097**	**0.52, (0.20 to 0.84), 0.008**	**0.68, (0.05 to 1.31), 0.076**
Diploma+	-0.14, (-0.67 to 0.39), 0.660	-0.03, (-0.24 to 0.17), 0.789	0.13, (-0.16 to 0.38), 0.391	0.19, (-0.21 to 0.59), 0.434	0.68, (-0.53 to 1.89), 0.353
BSC+	-0.81, (-5.69 to 4.34), 0.796	<0.01, (-0.25 to 0.25), 0.999	0.06, (-0.16 to 0.29), 0.635	**0.66, (0.32 to 1.00), 0.001**	0.58, (-0.09 to 1.26), 0.155
Job status					
Worker	Referent	Referent	Referent	Referent	Referent
Market	-0.61, (-1.80 to 0.59), 0.403	0.10, (-0.25 to 0.45), 0.629	0.03, (-0.38 to 0.44), 0.898	-0.07, (-0.47 to 0.34), 0.778	-0.39, (-4.04 to 3.26), 0.860
Service	0.40, (-0.06 to 0.87), 0.156	0.10, (-0.19 to 0.38), 0.548	-0.03, (-0.35 to 0.29), 0.870	-0.33, (-0.79 to 0.12), 0.228	-0.16, (-3.80 to 3.49), 0.943
Free Job	0.32, (-0.25 to 0.89), 0.355	0.14, (-0.08 to 0.35), 0.295	-0.03, (-0.29 to 0.23), 0.841	-0.17, (-0.47 to 0.12), 0.341	-0.11, (-3.81 to 3.60), 0.962
Government	-0.05, (-0.70 to 0.60), 0.898	-0.25, (-0.84 to 0.34), 0.481	-0.13, (-0.46 to 0.20), 0.523	**-0.40, (-0.78 to -0.02), 0.083**	0.11, (-3.53 to 3.76), 0.959
Income	0.16, (-0.01 to 0.31), 0.103	0.05, (-0.03 to 0.12), 0.306	-0.06, (-0.15 to 0.02), 0.208	**-0.14, (-0.23 to -0.05), 0.012**	-0.14, (-0.31 to 0.03), 0.181
House price	0.11, (-0.81 to 1.02), 0.848	0.07, (-0.03 to 0.16), 0.259	0.06, (-0.03 to 0.15), 0.246	-0.02, (-0.2 to 0.1), 0.817	-0.03, (-0.2 to 0.1), 0.733
Car price	-0.72, (-3.65 to 2.21), 0.685	0.16, (-0.06 to 0.39), 0.224	**0.35, (0.16 to 0.54), 0.002**	**0.32, (0.14 to 0.50), 0.004**	<0.01, (-0.16 to 0.16), 0.999
Hygiene cost	-0.20, (-0.48 to 0.07), 0.214	<0.01, (-0.12 to 0.12), 0.999	0.10, (-0.07 to 0.26), 0.344	0.12, (-0.01 to 0.25), 0.137	<0.01, (-0.18 to 0.18), 0.999
Motorcycling aim	0.22, (-0.21 to 0.66), 0.402	<0.01, (-0.15 to 0.15), 0.999	0.03, (-0.16 to 0.22), 0.785	-0.24, (-0.52 to 0.04), 0.164	-0.15, (-3.88 to 3.58), 0.948
Using helmet					
Always	Referent	Referent	Referent	Referent	Referent
Often	1.13, (-5.05 to 7.30), 0.764	**0.42, (0.01 to 0.84), 0.098**	0.33, (-0.11 to 0.78), 0.222	0.30, (-0.20 to 0.80), 0.327	-0.27, (-0.97 to 0.43), 0.523
Sometimes	1.68, (-4.48 to 7.85), 0.653	**0.76, (0.34 to 1.18), 0.003**	**0.58, (0.20 to 0.96), 0.013**	**0.51, (0.10 to 0.92), 0.041**	-0.11, (-1.12 to 0.89), 0.851
Seldom	1.64, (-4.53 to 7.85), 0.661	**0.69, (0.36 to 1.02), 0.001**	**0.52, (0.18 to 0.86), 0.013**	0.13, (-0.28 to 0.54), 0.604	-0.37, (-3.21 to 2.50), 0.831
Never	1.76, (-4.45 to 7.96), 0.641	**066, (0.32 to 0.99), 0.001**	**0.52, (0.18 to 0.85), 0.011**	0.10, (-0.49 to 0.68), 0.783	-0.22, (-0.92 to 0.48), 0.603
Having driving license	1.09, (-2.54 to 4.71), 0.621	0.24, (-0.07 to 0.55), 0.202	0.10, (-0.16 to 0.35), 0.545	-0.24, (-0.51 to 0.03), 0.143	-0.11, (-0.59 to 0.38), 0.718
Driving license period	-0.20, (-2.19 to 1.79), 0.867	-0.12, (-0.38 to 0.14), 0.439	-0.10, (-0.37 to 0.17), 0.558	-0.16, (-0.41 to 0.10), 0.307	-0.43, (-0.94 to 0.07), 0.158
Driving period	0.23, (-0.26 to 0.73), 0.437	-0.23, (-0.66 to 0.20), 0.378	**-0.78, (-1.10 to -0.46), <0.001**	**-0.95, (-1.26 to -0.65), <0.001**	**-0.58, (-1.03 to -0.13), 0.034**
Driving hour	0.28, (-0.23 to 0.78), 0.366	**0.45, (0.17 to 0.74), 0.009**	**0.52, (0.28 to 0.76), <0.001**	**0.42, (0.16 to 0.68), 0.009**	0.22, (-0.45 to 0.89), 0.590
Number of days used	0.18, (-0.13 to 0.49), 0.342	<0.01, (-0.10 to 0.10), 0.999	-0.06, (-0.24 to 0.11), 0.541	**-0.17, (-0.32 to -0.02), 0.065**	<0.01, (-0.26 to 0.26), 0.999
Sub-accident	0.92, (-1.77 to 3.61), 0.571	0.03, (-0.23 to 0.30), 0.837	-0.03, (-0.35 to 0.28), 0.868	-0.17, (-0.44 to 0.10), 0.303	-0.22, (-3.83 to 3.39), 0.920
Vehicle type					
Pedestrian	Referent	Referent	Referent	Referent	Referent
Motorcycle	**(-16.4 to -2.0), 0.037**	(-4.4 to 3.2), 0.789	(-1.4 to 0.5), 0.393	(-1.5 to 1.5), 0.972	(-0.1 to 2.9), 0.114
Car	(-1.4 to 1.3), 0.922	(-0.8 to 0.5), 0.687	(-0.3 to 0.8), 0.393	(-0.1 to 1.2), 0.139	(-5.1 to 7.6), 0.739
Lorry	(-2.3 to 0.1), 0.139	(-1.3 to 0.4), 0.373	(-0.8 to 0.4), 0.546	(-0.5 to 0.8), 0.732	(-0.5 to 0.6), 0.821
Cell phone answering				
Always	Referent	Referent	Referent	Referent	Referent
Often	-0.24, (-9.11 to 8.63), 0.964	-0.13 (-0.40 to 0.14), 0.424	-0.22, (-0.51 to 0.07), 0.206	**-0.87, (-1.39 to -0.34), 0.007**	-0.90, (-7.98 to 6.19), 0.835
Sometimes	-0.35, (-9.22 to 8.51), 0.947	-0.16, (-0.46 to 0.14), 0.373	-0.16, (-0.45 to 0.13), 0.371	**-0.80, (-1.38 to -0.22), 0.023**	-1.27, (-8.34 to 5.81), 0.768
Seldom	-0.51, (-9.36 to 8.34), 0.924	**-0.47, (-0.79 to -0.15), 0.017**	**-0.45, (-0.76 to -0.13), 0.021**	**-1.00, (-1.54 to -0.46), 0.003**	-1.40, (-8.51 to 5.71), 0.745
Never	-1.97, (-10.91 to 6.98), 0.717	**-0.81, (-1.11 to -0.52), <0.001**	**-0.81, (-1.13 to -0.50), <0.001**	**-0.80, (-1.43 to -0.17), 0.036**	-1.45, (-8.46 to 5.57), 0.734
ADHD score	**0.03, (0.01 to 0.04), 0.001**	**0.02, (0.01 to 0.02), <0.001**	**0.02, (0.01 to 0.03), <0.001**	**0.04, (0.02 to 0.05), <0.001**	0.02, (-0.01 to 0.05), 0.189
ASS score	**0.07, (0.02 to 0.11), 0.010**	**0.05, (0.04 to 0.07), <0.001**	**0.07, (0.04 to 0.09), <0.001**	**0.07, (0.03 to 0.12), 0.008**	0.02, (-0.06 to 0.11), 0.679
BSS score	**0.10, (0.06 to 0.13), <0.001**	**0.06, (0.04 to 0.07), <0.001**	**0.06, (0.04 to 0.08), <0.001**	**0.10, (0.06 to 0.14), <0.001**	**0.09, (0.04 to 0.13), 0.003**
CSS score	**0.04, (0.02 to 0.07), 0.002**	**0.03, (0.02 to 0.04), <0.001**	**0.04, (0.04 to 0.05), <0.001**	**0.05, (0.03 to 0.07), <0.001**	0.04, (-0.01 to 0.08), 0.142
DSS score	**0.06, (0.03 to 0.10), 0.004**	**0.04, (0.03 to 0.06), <0.001**	**0.05, (0.03 to 0.01), <0.001**	**0.07, (0.05 to 0.09), <0.001**	**0.04, (0.004 to 0.076), 0.068**

Abbreviations; B, Coefficient; CI, confidence interval; MRBQ, Motorcycle Rider Behavior Questionnaire; ADHD, Attention Deficit Hyperactivity Disorder; ASS score, subscale A measuring inattention; BSS score, subscale B measuring hyperactivity, impulsivity; CSS score, subscale C the sum of A and B subscales; DSS score, subscale D measuring ADHD index. Bold numbers represent the significant relationships.

**Table 4:** Estimates of coefficients of underlying predictors of MRBQ by LQR various modelling strategies

**Variables**	**Percentiles**				
	**5**	**25**	**50**	**75**	**95**
	**B (95 CI),** ***P***	**B (95 CI),** ***P***	**B (95 CI),** ***P***	**B (95 CI),** ***P***	**B (95 CI),** ***P***
**Model 1: Relationship between background variables and MRBQ Outcome variable**					
Driving period	**-0.75, (-1.19 to -.31), 0.001**	**-0.59, (-0.95 to -0.23), 0.001**	**-0.86, (-1.35 to -0.38), 0.001**	**-0.85, (-1.24 to -0.46), <0.001**	**-0.86, (-1.53 to -0.19), 0.012**
Hour driving	0.24, (-0.26 to 0.75), 0.346	**0.36, (0.04 to 0.68), 0.028**	0.07, (-0.22 to 0.37), 0.638	0.19, (-0.12 to 0.49), 0.231	**0.76, (0.14 to 1.38), 0.017**
Marital status (married)	0.10, (-0.19 to 0.40), 0.485	-0.03, (-0.31 to 0.25), 0.829	-0.05, (-0.29 to 0.20), 0.718	-0.06, (-0.37 to 0.24), 0.683	**-0.71, (-1.29 to -0.12), 0.018**
**Model 2: Relationship between background variables and ADHD total score with MRBQ Outcome variable**					
Driving period	**-0.47, (-0.90 to -.04), 0.032**	**-0.45, (-0.74 to -0.15), 0.003**	**-0.56, (-1.00 to -0.12), 0.012**	**-0.64, (-1.06 to -0.23), 0.003**	**-0.77, (-1.45 to -0.09), 0.027**
Hour driving	0.23, (-0.20 to 0.65), 0.296	**0.40, (0.10 to 0.70), 0.008**	0.015, (-0.10 to 0.41), 0.241	0.15, (-0.12 to 0.42), 0.269	**0.71, (0.13 to 1.28), 0.016**
ADHD score	**0.02, (0.01 to 0.03), <0.001**	**0.01, (0.01 to 0.02), <0.001**	**0.02, (0.01 to 0.03), <0.001**	**0.02, (0.01 to 0.03), 0.006**	0.01, (-0.02 to 0.04), 0.521
Marital status (married)	0.26, (-0.04 to 0.56), 0.090	-0.03, (-0.26 to 0.20), 0.799	0.04, (-0.20 to 0.29), 0.724	-0.08, (-0.39 to 0.23), 0.617	**-0.73, (-1.33 to -0.13), 0.017**
**Model 3: Relationship between background variables and ADHD ASS and BBS subscale scores with MRBQ Outcome variable**					
Driving period	**-0.44, (-0.79 to -.09), 0.014**	**-0.42, (-0.70 to -0.13), 0004**	**-0.62, (-1.06 to -0.18), 0.006**	**-0.72, (-1.13 to -0.31), 0.001**	-0.56, (-1.29 to 0.18), 0.136
Hour driving	0.29, (-0.14 to 0.71), 0.183	**0.48, (0.18 to 0.78), 0.002**	0.18, (-0.12 to 0.48), 0.236	0.18, (-0.11 to 0.47), 0.223	0.20, (-0.46 to 0.86), 0.598
ASS score	**0.07, (0.01 to 0.14), 0.046**	0.03, (0.01 to 0.06), 0.109	0.01, (-0.04 to 0.05), 0.739	-0.01, (-0.06 to 0.04), 0.740	-0.04, (-0.19 to 0.11), 598
BSS score	-0.01, (-.07 to 0.07), 0.929	0.02, (-0.02 to 0.06), 0.338	0.04, (-0.01 to 0.08), 0.060	**0.07, (0.02 to 0.12), 0.008**	**0.10, (0.01 to 0.19), 0.036**
**Model 4: Relationship between background variables and ADHD DDS subscale scores with MRBQ Outcome variable**					
Driving period	**-0.57, (-1.02 to -.12), 0.013**	**-0.43, (-0.75 to -0.11), 0.009**	**-0.63, (-1.07 to -0.19), 0.005**	**-0.65, (-1.08 to -0.23), 0.003**	**-0.76, (-1.38 to -0.13), 0.018**
Hour driving	0.27, (-0.17 to 0.71), 0.224	0.31, (-0.01 to 0.62), 0.053	0.16, (-0.09 to 0.40), 0.206	0.12, (-0.17 to 0.40), 0.417	**0.64, (0.07 to 1.22), 0.028**
Marital status (married)	0.15, (-0.16 to 0.45), 0.342	0.01, (-0.24 to 0.25), 0.987	-0.01, (-0.25 to 0.22), 0.906	-0.10, (-0.39 to 0.19), 0.491	**-0.97, (-1.23 to -0.11), 0.020**
DSS score	**0.04, (0.01 to 0.06), 0.011**	**0.03, (0.02 to 0.05), <0.001**	**0.04, (0.01 to 0.06), <0.001**	**0.04, (0.01 to 0.07), 0.006**	0.03, (-0.03 to 0.09), 0.317

Abbreviations: B, Coefficient; CI, confidence interval; MRBQ, Motorcycle Rider Behavior Questionnaire; ADHD, Attention Deficit Hyperactivity Disorder; ASS score, subscale A measuring inattention; BSS score, subscale B measuring hyperactivity, impulsivity; CSS score, subscale C the sum of A and B subscales; DSS score, subscale D measuring ADHD index. Bold numbers represent the significant relationships.

## References

[1] WHO. Global status report on road safety 2013. Available from: http://www.who.int/violence_injury_prevention/road_safety_status/2013/en/. Accessed May 20, 2017.

[2] Alinia S, Khankeh H, Maddah SS, Negarandeh R (2015). Barriers of pre-hospital services in road traffic injuries in Tehran: the viewpoint of service providers. Int J Community Based Nurs Midwifery.

[3] Rad M, Martiniuk AL, Ansari-Moghaddam A, Mohammadi M, Rashedi F, Ghasemi A (2016). The pattern of road traffic crashes in south East Iran. Glob J Health Sci.

[4] Ghorbani A. Nabavi Fard H, Khoshhal M, Hosseini H. Costs imposed on the effects of mortality due to traffic accidents. Traffic Management Studies 2011;20:49-58. [Persian].

[5] Mahdian M, Sehat M, Fazel MR, Moraveji A, Mohammadzadeh M (2015). Epidemiology of Urban traffic accident victims hospitalized more than 24 hours in a level III trauma center, Kashan county, Iran, during 2012-2013. Arch Trauma Res.

[6] Ghadirzadeh MR, Fadaye Vatan R, Akbari kamrani AA, Davatgaran K, Hashemi Nazari SS, Mirtorabi SD (2012). Road accident mortality rate of the Iranian elderly from 2006 to 2008. Iranian Journal of Ageing.

[7] Peden M, Scurfield R, Sleet D, Mohan D, Hyder AA, Jarawan E (2004). World report on road traffic injury prevention.

[8] Barros AJ, Amaral RL, Oliveira MS, Lima SC, Goncalves EV (2003). Traffic accidents resulting in injuries: underreporting, characteristics, and case fatality rate. Cad Saude Publica.

[9] Horswill MS, Helman S (2003). A behavioral comparison between motorcyclists and a matched group of non-motorcycling car drivers: factors influencing accident risk. Accid Anal Prev.

[10] Graziano PA, Reid A, Slavec J, Paneto A, McNamara JP, Geffken GR (2015). ADHD symptomatology and risky health, driving, and financial behaviors in college: the mediating role of sensation seeking and effortful control. J Atten Disord.

[11] Naghavi M, Jafari N, Alaeddini F, Akbari M (2004). Epidemiology of injuries due to external causes in the Islamic Republic of Iran.

[12] Evans L (1996). The dominant role of driver behavior in traffic safety. Am J Public Health.

[13] Kopits E, Cropper M (2005). Traffic fatalities and economic growth. Accid Anal Prev.

[14] Ranney TA, Mazzae E, Garrott R, Goodman MJ. NHTSA driver distraction research: Past, present, and future. Driver distraction internet forum; 2000.

[15] Motevalian SA, Asadi-Lari M, Rahimi H, Eftekhar M (2011). Validation of a persian version of motorcycle rider behavior questionnaire. Ann Adv Automot Med.

[16] Motevalian A. Questionnaire validation motorcyclists driving behavior [dissertation]. Tehran: Iran University of Medical Sciences; 2010. [Persian].

[17] Shappell SA, Wiegmann DA. A human error approach to aviation accident analysis: the human factors analysis and classification system. Burlington: Ashgate Publishing Ltd; 2012.

[18] Weiss G, Hechtman L, Perlman T, Hopkins J, Wener A (1979). Hyperactives as young adults: a controlled prospective ten-year follow-up of 75 children. Arch Gen Psychiatry.

[19] Barkley RA, Guevremont DC, Anastopoulos AD, DuPaul GJ, Shelton TL (1993). Driving-related risks and outcomes of attention deficit hyperactivity disorder in adolescents and young adults: a 3- to 5-year follow-up survey. Pediatrics.

[20] Sadeghi-Bazargani H, Abedi L, Mahini M, Amiri S, Khorasani-Zavareh D (2015). Adult attention-deficit hyperactivity disorder, risky behaviors, and motorcycle injuries: a case-control study. Neuropsychiatr Dis Treat.

[21] Anderson JA. An Introduction to Neural Networks. Massachusetts, MA: Institute of Technology; 1996.

[22] Sakashita C, Senserrick T, Lo S, Boufous S, Rome LD, Ivers R (2014). The Motorcycle Rider Behavior Questionnaire: Psychometric properties and application amongst novice riders in Australia. Transportation Research Part F: Traffic Psychology and Behaviour.

[23] Safiri S, Sadeghi-Bazargani H, Amiri S, Khanjani N, Safarpour H, Karamzad N (2013). Association between Adult attention deficit-hyperactivity disorder and motorcycle traffic injuries in Kerman, Iran: a case-control study. Journal of Clinical Research & Governance.

[24] Bottai M, Cai B, McKeown RE (2010). Logistic quantile regression for bounded outcomes. Stat Med.

[25] Elliott MA, Baughan CJ, Sexton BF (2007). Errors and violations in relation to motorcyclists’ crash risk. Accid Anal Prev.

[26] Ozkan T, Lajunen T, Dogruyol B, Yildirim Z, Coymak A (2012). Motorcycle accidents, rider behaviour, and psychological models. Accid Anal Prev.

[27] Sadeghi-Bazargani H, Amiri S, Hamraz S, Malek A, Abdi S, Shahrokhi H (2014). Validity and reliability of the Persian version of Conner’s adult ADHD rating scales : observer and self-report screening versions. Journal of Clinical Research & Governance.

[28] DSMV 5. Avaiable form: https://www.psychiatry.org/File%20Library/Psychiatrists/Practice/DSM/APA_DSM_Changes_from_DSM-IV-TR_-to_DSM-5.pdf. Accessed May 16, 2015.

[29] Orsini N, Bottai M (2011). Logistic quantile regression in Stata. Stata J.

[30] Coopersmith HG, Korner-Bitensky NA, Mayo NE (1989). Determining medical fitness to drive: physicians’ responsibilities in Canada. CMAJ.

[31] Watson BC, Tunnicliff DJ, White KM, Schonfeld CC, Wishart DE. Psychological and social factors influencing, motorcycle rider intentions and behaviour. Canberra, ACT: Australian Transport Safety Bureau; 2007.

[32] Columbu S, Bottai M. Cognitive deterioration in cancer patients using logistic quantile regression. Available from: http://meetings.sis-statistica.org/index.php/sis2013/ALV/paper/view/2577/333. Accessed May 20, 2017.

[33] Feizi A, Aliyari R, Roohafza H (2012). Association of perceived stress with stressful life events, lifestyle and sociodemographic factors: a large-scale community-based study using logistic quantile regression. Comput Math Methods Med.

[34] Agresti A, Kateri M. Categorical Data Analysis. In: Lovric M, ed. International Encyclopedia of Statistical Science. Berlin, Heidelberg: Springer; 2011. p. 206-8.

[35] Simas AB, Barreto-Souza W, Rocha AV (2010). Improved estimators for a general class of beta regression models. Comput Stat Data Anal.

[36] Schmid M, Wickler F, Maloney KO, Mitchell R, Fenske N, Mayr A (2013). Boosted beta regression. PLoS One.

